# Role of tumor microenvironment in ovarian cancer pathobiology

**DOI:** 10.18632/oncotarget.25126

**Published:** 2018-04-27

**Authors:** Alia Ghoneum, Hesham Afify, Ziyan Salih, Michael Kelly, Neveen Said

**Affiliations:** ^1^ Department of Cancer Biology, Wake Forest University School of Medicine, Winston Salem, NC 27157, USA; ^2^ Department of Cancer Pathology, Wake Forest University School of Medicine, Winston Salem, NC 27157, USA; ^3^ Department of Cancer Obstetrics and Gynecology, Wake Forest University School of Medicine, Winston Salem, NC 27157, USA; ^4^ Department of Cancer Urology, Wake Forest University School of Medicine, Winston Salem, NC 27157, USA

**Keywords:** ovarian cancer, tumor microenvironment, peritoneal metastasis, targeted therapy, resistance and recurrence

## Abstract

Ovarian cancer is the fifth most common cancer affecting the female population and at present, stands as the most lethal gynecologic malignancy. Poor prognosis and low five-year survival rate are attributed to nonspecific symptoms and below par diagnostic criteria at early phases along with a lack of effective treatment at advanced stages. It is thus of utmost importance to understand ovarian carcinoma through several lenses including its molecular pathogenesis, epidemiology, histological subtypes, hereditary factors, diagnostic approaches and methods of treatment. Above all, it is crucial to dissect the role that the unique peritoneal tumor microenvironment plays in ovarian cancer progression and metastasis. This review seeks to highlight several important aspects of ovarian cancer pathobiology as a means to provide the necessary background to approach ovarian malignancies in the future.

## INTRODUCTION

Several aspects of ovarian cancer (OvCa) deem it a very challenging malignancy to diagnose and treat. Even though the five-year survival rate is high when women are diagnosed in the early stages of OvCa, most women are rarely diagnosed in early stages as the symptoms are merely vague abdominal discomfort or bloating [1]. The five-year survival rate of women diagnosed with OvCa drops precipitously when women are diagnosed with stage III or IV as aggressive metastasis to neighboring abdominal organs has already progressed [1, 2]. Patients’ survival is strongly correlated with the outcome of the surgical debulking [3]. The standard clinical management for advanced stage OvCa includes debulking surgery followed by adjuvant chemotherapy or neoadjuvant chemotherapy followed by surgery in patients who present with un-resectable disease [4]. However, after initial response, tumor recurrence from residual disease is encountered in about 70% of patients who will eventually die of a progressively chemo-resistant cancer [4]. Indeed, optimal surgical debulking of tumors (< 1 cm of residual tumor) significantly improves patients’ survival compared to sub-optimal debulking [4]. Unfortunately, suboptimal debulking (>1 cm of residual tumor) is frequently encountered due to widespread microscopic and inaccessible lesions throughout the abdomen preventing complete removal of the tumor [4]. There remains a need for improved OvCa treatment that addresses the current limitations of surgical debulking and reduces treatment resistance that often arises in response to chemotherapy [4].

OvCa is heterogeneous and affected by epigenetic and genetic factors [5]. A major reason for the lack of success in effectively eradicating OvCa can be due to the complex interconnected signaling networks coupled with the distinctive peritoneal tumor microenvironment (TME) [6]. Several immune cells, including tumor associated macrophages (TAMs), T cells, natural killer (NK) cells in addition to fibroblasts and a wide host of chemokines and cytokines all interact with each other to promote the growth and metastasis of OvCa cells [7]. Therefore, understanding the pathobiology of OvCa and its unique TME that hosts this malignancy, is crucial in our development of more sensitive diagnostic tools and enhanced treatment options.

### Epidemiology

Epithelial OvCa (EOC) is the leading cause of gynecologic cancer-related death in the USA. More than 70% of patients are diagnosed with advanced disease (Stage III or IV) [4, 8]. In the year 2017 alone, a growing 22,240 new cases and 14,080 cancer-related deaths occured in USA [9]. The incidence is higher in Caucasians than in Hispanics, American Indian/Alaska Natives, Blacks, and Asian/Pacific Islanders [10]. The mean age of diagnosis of ovarian cancer is 63 years. Differences in age distribution are summarized in (http://seer.cancer.gov/statfacts/html/ovary.html).

### Risk factors

#### Genetic risk factors

The lifetime risk of developing OvCa in the USA is currently 1.4%. The risk increases with mutations in several genes namely, BRCA1/2, mismatch repair and ARID1 genes [11]. Women with BRCA mutations have an increased risk of developing ovarian, fallopian tube and peritoneal cancer, specifically, 20–50% in BRCA1 and 10–20% in BRCA2 [12]. Women with mutations in mismatch repair genes associated with type 2 Lynch syndrome are at higher risk of developing colon, endometrial and ovarian cancers [13, 14]. Similarly, women with mutations in ARID1A are at risk of developing endometrial or clear cell ovarian carcinoma [11].

#### Non-genetic risk factors

Include incessant ovulation as well as repeated rupture and repair of ovarian follicles with continued exposure to gonadotropins [15]. Nulliparity and infertility, both interfere with protective hormone release, are considered risk factors. In support of this, multiparous women who are pregnant after the age of 35 years are at a reduced risk of developing EOC [16–18]. Gynecologic diseases such as endometriosis, polycystic ovary syndrome (PCOS), and postmenopausal hormonal therapy perturb the estrogen and progesterone cycle and increase the risk of OvCa [19, 20]. Intrauterine devices, obesity, cigarette smoking, exposure to talc and asbestos have also been shown to increase the risk of OvCa [21–24].

#### Protective factors

Include oral contraceptives, multiparity, salpingo-oophrectomy, tubal ligation, hysterectomy, breast feeding, nonsteroidal anti-inflammatory (NSAID) drugs and acetaminophen.

#### Pathological subtypes

Many pathological subtypes are described for EOC. These are further classified into serous, clear cells, mucinous, and endometrioid.

#### Serous cancer

High-grade serous cancer (HGSC) compromise 70 to 80% of all cases and typically arises from either the surface of the ovary or from the distal fallopian tube [25]. HGSC strongly and diffusely expresses p53 and p16. HGSC also expresses Wilm's tumor-1 (WT-1), estrogen receptor (ER), and Paired Box-8 (PAX-8) in most cases as well as a high Ki67 proliferative index. Genetic alterations in BRCA1 or BRCA2 germline mutations are present in up to 10% of women with HGSC. Additional associated genetic alterations that are specific to HGSC are *TP53 (as high as 96%)*, *NF1, RB1, CDK12, PTEN* and *PIK3CA* [5, 26, 27].

Low-grade serous cancer (LGSC) comprise less than 5% of OvCa. LGSC has a low Ki67 proliferative rate with normal p53 expression, and commonly expresses WT-1, ER, and progesterone receptor (PR). Mutations in *BRAF* and *KRAS* typically lead to LGSC [2, 28].

#### Endometrioid carcinoma

Comprises 10% of epithelial types. Cancer cells express vimentin, ER, PR, PAX-8, and CA125. Genetic mutations of *CTNNB-1* (β-catenin), *PTEN*, *PIK3CA* and *ARID1A* with microsatellite instability are also present [29, 30]. Endometrioid carcinoma as with clear cell carcinoma, typically arise from endometriosis which is linked to the theory of retrograde menstruation [25].

#### Clear cell carcinoma

Comprises 10% of epithelial cases. Hypoxia-inducible factor 1 alpha (HIF-1 α) [31], glypican-3 [32], and hepatocyte nuclear factor 1-beta (HNF-1 beta) are highly expressed [33]. Genetic alterations include mutations in *ARID1A KRAS, PTEN,* and *PIK3CA* [34].

#### Mucinous carcinoma

Comprises 3% of epithelial cases. Gastrointestinal markers CK20, CDX2, CK7 and molecular mutations as *KRAS* as well as mucin genes *MUC2, MUC3*, and *MUC17* are commonly expressed [35, 36].

### Molecular pathogenesis

Tumors of the ovary have been divided into type I which includes LGSC, mucinous, low grade endometrioid and clear cell carcinoma and type II which includes HGSC, high grade endometrioid and undifferentiated/malignant mixed carcinomas. Type I tumors include mutations in *KRAS, BRAF, PTEN* and *CTNNB-1,* while type II tumors are typically associated with *p53* mutations. However, evidence from mouse models of OvCa indicated that p53 mutations alone are not sufficient to drive invasive carcinoma. As HGSC composes 70% of all ovarian serous tumors, its pathogenesis is especially important [35].

The pathogenesis of OvCa can also be segregated by anatomical origins namely ovarian or tubal (fallopian tube) derived [37]. The ovarian subtypes arise from ovarian surface epithelia which are derived from mesothelial coelomic epithelia covering the ovaries. They are often a result of the repeated formation of cortical inclusion cysts and endometriosis. It is hypothesized that ovarian carcinoma of this subtype arises from ovarian inclusions cysts that underwent Mullerian metaplasia [38]. The Fallopian or tubal subtypes originate from the coelom, namely the Mullerian or paramesonephric ducts. The majority of serous tumors appear to arise from the secretory cells in the distal fallopian tube. Serous tubal intraepithelial cancer (STIC) is the precursor lesion with *TP53* mutations similar to those found in HGSC [39–41]. More recent studies theorize that the exact origin of tubal subtypes is at the junctional zone between the fallopian tube epithelium and the mesothelium of the tubal serosa. This site in particular, confers extensive connections with the lymphatic system making it relatively easy to invade and metastasize to the abdominal cavity. Thus, prophylactic salpingectomy is encouraged for women undergoing hysterectomy for benign conditions [42].

### Clinical features

OvCa can manifest as either acute or subacute in nature. Acute forms are usually due to advanced stages of cancer spread leading to bowel obstruction and pleural effusion. Subacute cases will manifest as a unilateral or bilateral adnexal mass, pelvic pain or abdominal pain, postmenopausal bleeding, rectal bleeding or atypical glandular cells on cervical cytology [1, 43].

### Diagnosis of EOC

Diagnostic studies are categorized into two phases: an initial evaluation of the presumed adnexal or abdominal mass by imaging studies such as abdominal ultrasound and MRI followed by surgical evaluation, and pathological identification of the subtype, grading, and staging. Pathological grading and identification of the subtypes are achieved after percutaneous fine needle biopsy or after cytoreduction [44]. Several tumor markers are considered in the diagnosis such as CA125 (cancer antigen 125), human epididymis protein 4 (HE4) and carcinoembryonic antigen (CEA) that are increased in advanced EOC and are considered as prognostic markers, though they lack specificity and sensitivity [44].

### Determinants of peritoneal metastasis

#### Cancer cells

EOC is unique among cancers in that cancer cells have diverse progenitors, ovarian surface epithelium (OSE) and fimbrial epithelia, that express common epithelial markers as keratins, EpCAM and E-cadherin as well as mesenchymal markers as vimentin and N-cadherin [45–47]. Malignant cells are shed from the primary tumor into the peritoneal cavity where they survive as free-floating single cells or aggregate as spheroids in the peritoneal fluid “malignant ascites”. Single cells and spheroids can not only survive anchorage-independent apoptosis “anoikis”, but also can proliferate in suspension and seed onto the mesothelial cells lining the peritoneal cavity resulting in extensive peritoneal dissemination [48, 49]. Phenotypic characterization of single and multicellular malignant cells isolated from ascitic fluid revealed that these cells exhibit dual “hybrid” as well as heterogeneous E-and N-Cadherin expression [49]. The latter study also reported cadherin-dependent diversity in cell-cell interactions, spheroid formation, and ultrastructure. This is further supported by an elegant report from the same group [50] implicating cadherin-plasticity in mesothelial adhesion, clearance and collagen invasion. Cadherin plasticity is also implicated in the dynamic switch between epithelial-mesenchymal transition (EMT) and mesenchymal-epithelial transition (MET). EMT-MET switch is regulated by complex sequential transcriptional machinery with early induction of the transcription factors SNAIL (SNAI1) and slug; whereas SNAI2, ZEB1/2 and TWIST were induced at later phases [51–55]. EMT- transcription factors are induced by a plethora of upstream factors that act individually or synergistically to induce an invasive phenotype of EOC cells. The expression of EMT-inducing transcription factors (Snail, Slug, Twist and Zeb1/2) is associated with metastatic, recurrent and chemo-resistant tumors and poor prognosis [51, 53, 55–58]. Correlation between EMT and aggressiveness of OvCa is supported by the downregulation of E-cadherin [59] and overexpression of mesenchymal signatures specifically transforming growth factor beta and its receptors (TGFβ/TGFβRs), CD44 [60], bone morphogenetic proteins and their receptors (BMPs/BMPRs), receptor tyrosine kinases and their ligands [54], Wnt [61, 62] and Notch [53] signaling pathways. In addition to intrinsic EMT inducers activated in cancer cells, cues from the peritoneal TME strongly induce EMT. For example, mesothelial cells, adipocytes and ascitic fluid rich in growth factors, bioactive lipids, matrix metalloproteases (MMPs), as well as inflammatory and immune cells; all induce hypoxia, inflammation and oxidative stress and corroborate to induce EMT [63–66].

#### Mesothelial cells

Mesothelial cell**s** are the first barrier that faces metastatic OvCa cells. They are organized as a single layer of simple epithelium covering the sub extracellular matrix (ECM). rich in collagen I that covers abdominal, pelvic as well as visceral organs including the omentum [67–69]. Apically, mesothelial cells secrete glycosaminoglycans, surfactant and proteoglycans to establish an anti-adhesive surface. The bidirectional cross-talk between cancer and mesothelial Cells promotes cancer cell chemotaxis to the mesothelial cells, followed by integrin-mediated adhesion and invasion with subsequent increase in MMPs, and urokinase type plasminogen activator (uPA) and its receptor (uPAR) [70–73]; eventually leading to mesothelial clearance and invasion of the sub-mesothelial layers [74–76]. In addition, clinical reports show that cancer cells preferentially bind to regions of disrupted mesothelium at sites of entry of lymphatic and blood vessels [67–69, 77].

The propensity of OvCa to metastasize to the mesothelial cells is initially instigated by cancer cell secretome that preconditions the mesothelial cell niche to induce the expression of multiple pro-inflammatory mediators as bioactive lipids (e.g. LPA)/inflammatory cytokines/chemokines [78–80], ECM/integrins [67, 68, 81–86], cell adhesion molecules as VCAM1, ICAM1, CD44/HA [87–89], and uPA/uPAR [71, 90]. OvCa cell adhesion to mesothelial cells is mediated by bidirectional binding of ECM, integrins and cell adhesion molecules. This binding activates multiple downstream signaling pathways that in corroboration with activation of oncogenic signaling pathways, promote cancer cell colonization, invasiveness, and metastasis.

#### The omentum and omental adipocytes

**Figure 1:**
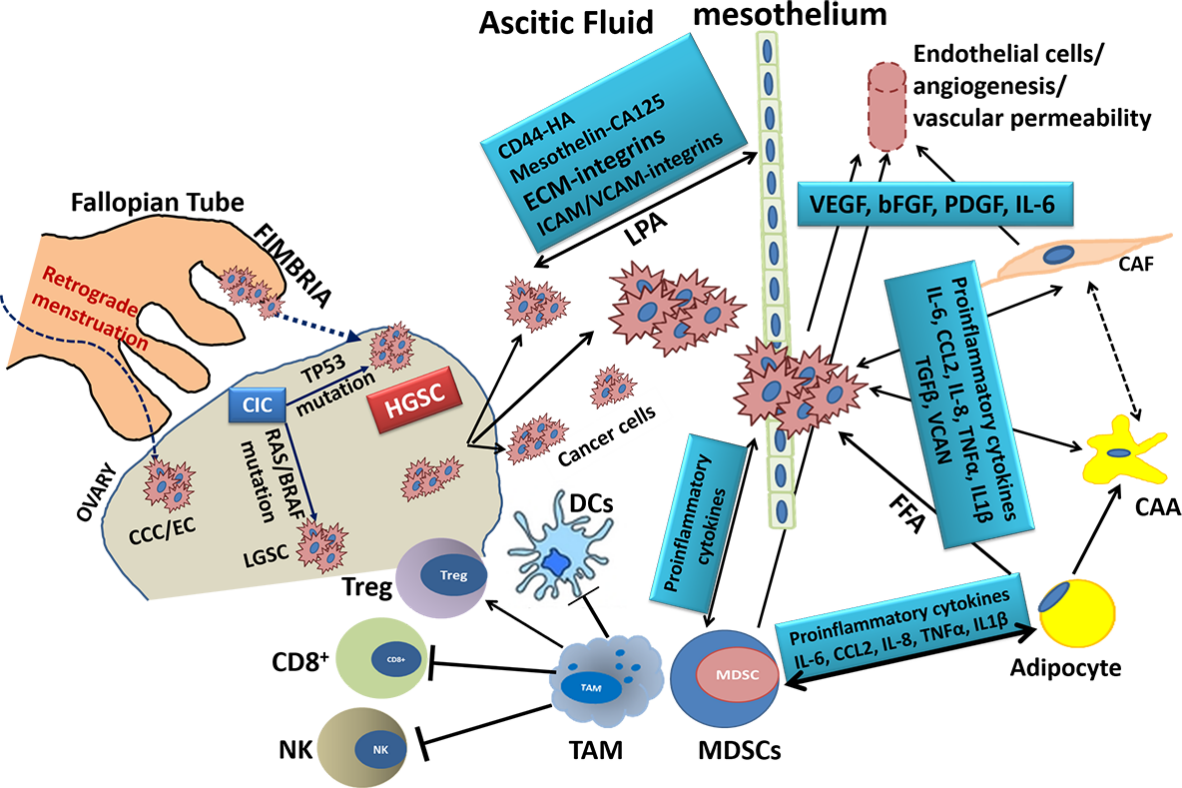
Schematic Representation of the key cell types in Ovarian Cancer Microenvironment and the molecules involved in their interactions HGSC: high grade serous cancer; LGSC: low grade serous cancer; CCC: clear cell carcinoma; EC: endometrial carcinoma; CIC: carcinoma *in situ*; CAA: cancer-associated adipocyte; CAF: cancer-associated fibroblast; FFA: free fatty acids; VEGF: vascular endothelial growth factor; bFGF: basic fibroblast growth factor; PDGF: platelet-derived growth factor; VCAN: versican; CD8+, cytotoxic T cell; Treg: regulatory T cell; DCs: dendritic cells; MDSCs: myeloid-derived suppressor cell; ECM, extracellular matrix; IL-x, interleukin-x; ICAM/VCAM: intercellular/vascular adhesion molecule; HA: hyaluronic acid; CA125: cancer antigen 125; LPA: lysophosphatidic acid; NK: natural killer cell; TAM: tumor-associated macrophage; TGFβ: growth transforming growth factor β; TNFα: tumor necrosis factor-α.

The omentum, which is subdivided into lesser and greater, is a double layered peritoneal fold that covers the intestines and abdominal organs. Physiologically, it functions as a fat and energy depot due to the abundance of white adipocytes [91, 92]. It also has a role in immune surveillance via aggregates of macrophages known as milky spots that play an important role in containing intraperitoneal infections [93, 94]. Milky spots also play an important role in the tropism of OvCa cells to the omentum [95–97]. Importantly, omental adipocytes release cytokines/chemokines “adipokines”, which contribute to OvCa cell homing, invasion and metastasis [91, 92, 95, 98]. The bidirectional interaction between omental adipocytes and cancer cells causes dedifferentiation and reprogramming of adipocytes into cancer-associated adipocytes (CAA) [98]. In this process, cancer cells secrete cytokines and chemokines that induce lipolysis in adipocytes, breaking their lipids (triglycerides), and releasing fatty acids and glycerol. Consequently, adipocytes undergo de-differentiation into a pre-adipocyte stage (fibroblastoid) and secrete adipokines [91, 98] (Figure 1). In turn, the uptake of fatty acids by cancer cells increases where they are used for generation of energy by beta oxidation [91] to meet the increasing demands of the rapidly proliferating OvCa cells.

#### Fibroblasts

Cancer associated fibroblasts (CAFs) play an important role in the EOC progression. In the peritoneal milieu, the origin of CAFs is unclear. The activation of resident fibroblasts and mesenchymal stem cells has long been considered the main origin of CAFs in the tumor microenvironment [99]. However, mesothelial cells have been shown as an important source of activated fibroblasts in inflammatory and fibrotic peritoneal pathologies as peritoneal dialysis, in which mesothelial cells are converted into myofibroblasts through mesothelial to mesenchymal transition (MMT) [100]. This hypothesis was supported in clinical specimens from patients with peritoneal metastases from ovarian and colon cancers, in which submesothelial fibroblasts expressing both mesothelial (calretinin, cytokeratins, mesothelin) and myofibroblasts (α-SMA) markers were detected by immunostaining [101]. More recently, mechanistic studies by Rynne-Vidal and colleagues [102] demonstrated that mesothelial cells isolated from ascitic fluid of OvCa patients with peritoneal metastases underwent MMT and promoted *in vivo* growth of xenografts through TGF-β-Smad-dependent MMT program, highlighting the crucial impact of the TGF-β-mediated bidirectional communication between OvCa cells and mesothelial cell-derived CAFs to form a suitable metastatic niche [102].

Another source of CAFs in the unique peritoneal TME is the omental adipocytes that have undergone delipidation and de-differentiation into pre-adipocyte fibroblastoid or stem-like stage [98, 103, 104]. Although this hypothesis has not yet been reported in OvCa, our unpublished data as well as reports in breast and pancreatic cancer [98, 103, 104] support its implication in OvCa. This is further supported by earlier reports of adipocyte de-differentiation into fibroblasts in inflammatory fibrotic changes encountered in dysfunctional adipose tissues in obesity and type 2 diabetes [105]. Moreover, endothelial to mesenchymal transition that has been reported in vasculopathies and atherosclerotic plaques [106] was suggested as a source of CAFs in OvCa [107].

**Figure 2:**
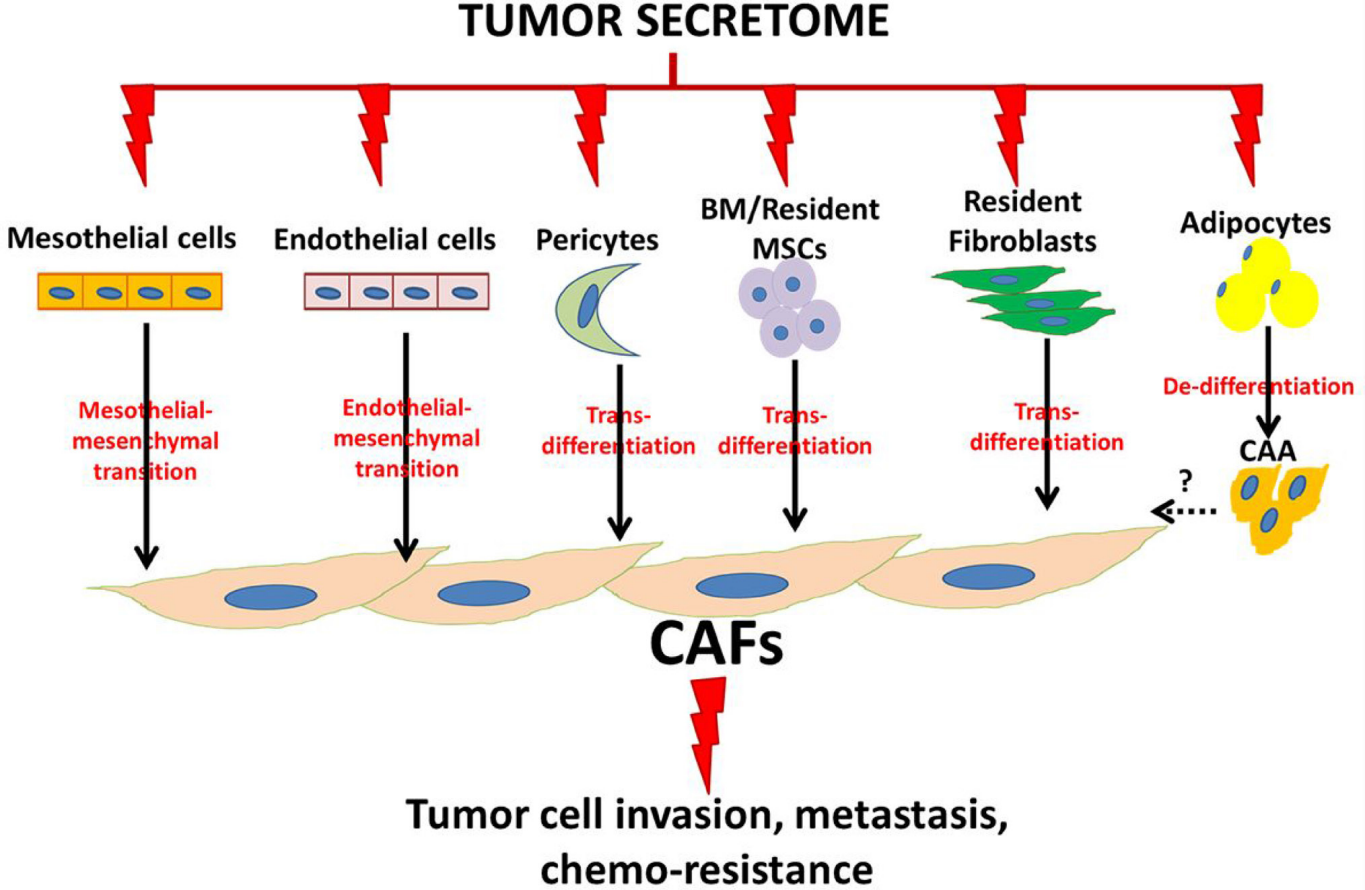
Schematic illustration of ovarian cancer cell-induced phenotypic commitment of stromal cells into cancer-associated phenotype CAFs: cancer-associated fibroblasts, CAA: cancer-associated adipocytes, BM: bone marrow, MSCs: mesenchymal stem cells.

The CAF phenotype is induced by environmental cues (Figure 2) characterized by inflammation, and hypoxia activating fibroblasts to exhibit characteristics of both myofibroblasts and secretory phenotype further contributing to inflammatory TME, cancer invasiveness and metastasis [101, 102, 108–111]. Increased number of CAFs corresponds with a more advanced OvCa stage, higher frequency of lymph node metastases, and amplified lymphatic and micro-vessel density [111]. CAFs can be activated by multiple mechanisms triggered by secreted factors from OvCa cells as TGF-β1, inflammatory cytokines and chemokines, reactive oxygen species (ROS) as well as MMPs [108]. The activation of CAF by TNFα has been shown to upregulate transforming growth factor α (TGF-α) through an inflammatory process activating NFkB. In turn CAF-derived TGFα induces epidermal growth factor (EGFR) signaling in cancer cells which stimulates cancer cell growth [112]. CAFs can also be activated through increased expression of progranulin (PGRN) peptide that stimulates EMT in cancer cells and upregulates the expression of smooth muscle actin α (α-SMA) in fibroblasts. High levels of both PGRN and α-SMA and low E-cadherin levels were associated with poor prognosis [108, 113]. Importantly, the molecular cross-talk between cancer cells and CAFs in the ovarian TME has been shown to be regulated by TGFβ/TGFβRs/SMAD pathway in CAFs with subsequent overexpression and secretion of target genes as versican [110]. The latter mediates tumor migration and invasion through binding to CD44 with subsequent activation of the NFkB and JNK signaling pathways in OvCa cells further supporting a pro-inflammatory TME and tumor progression [110].

#### Tumor associated macrophages (TAMs)

The pro-inflammatory peritoneal TME characterized by increased LPA is continuously produced by cancer and mesothelial cells with subsequent increase in several pro-inflammatory cytokines and chemokines including macrophage chemoattractant protein-1 (MCP-1/CCL2) which recruits macrophages to the peritoneal TME promoting tumor growth, invasiveness, vascular permeability and angiogenesis. The cross-talk between cancer cells and TAMs upregulates the secretion of MMPs, uPA/uPAR, and prostaglandin E2 (PGE2) [79, 80, 114–117] which influence tumor migration and invasion through activation of NFkB, the key regulator of pro-inflammatory molecules in both TAMs and cancer cells, making them even more important in tumor progression [79, 80]. In addition, the increased infiltration of TAMs in the peritoneal TME not only promotes cancer cell invasiveness but also contributes to an immunosuppressive environment that suppresses the function of T cells, dendritic cells (DCs) and natural killer (NK) cells [7]. TAMs also contribute to the phenotypic switch of fibroblasts into cancer associated fibroblasts (CAFs), and in turn activate multiple pathways specifically TGFβ pathway that leads to inflammation and immune suppression, and the associated chemo-resistance, recurrence and poor prognosis [118, 119]. In addition, increased TAMs in the OvCa milieu was found to decrease the sensitivity to VEGF targeting therapy [120]. The augmented inflammatory TME promoted many clinical trials targeting inflammatory cytokines/chemokines and their receptors, TGFβ/TGFβRs as well as COX-2 inhibitors (Table 1).

**Table 1:** Clinical trials targeting the ovarian cancer cells and their interactions with tumor microenvironment

Drug	Target	Clinical Trial	NCT Trial
Aflibercept (VEGF trap)	vascular endothelial growth factor (VEGF)	Phase 2	NCT00327171 NCT00327444 NCT00396591
Bevacizumab + paclitaxel and carboplatin	VEGF-A	Phase 3	NCT01239732
Bevacizumab and Erlotinib	VEGF-A + EGFR	Phase 2	NCT00130520
Bevacizumab + Carboplatin	VEGF-A	Phase 2	NCT00937560 NCT00744718
Chiauranib	serine-threonine kinases: aurora kinase B (aurora B), VEGF receptors (VEGFRs), stem cell factor receptor (c-KIT), and platelet-derived growth factor receptors (PDGFRs)	Phase 1 Phase 2	NCT03166891
Nintedanib + Bevacizumab	VEGFR1/2/3, FGFR1/2/3 and PDGFRα/β (angiogenesis and fibrosis)	Phase 1	NCT02835833
INCB062079	fibroblast growth factor receptor 4 (FGFR4)	Phase 1	NCT03144661
Sorafinib + paclitaxel and carboplatin	Multi-targeted Receptor Tyrosine Kinase Inhibitor (RTKi)	Phase 2	NCT00390611
Sunitinib (SU11248)	Multi-targeted RTKi	Phase 2	NCT00543049 NCT00768144 NCT00453310
Tocilizumab and Interferon alpha 2-b (IFN-α2b)+ Carboplatin and Caelyx or doxorubicin	Interleukin-6 receptor (IL-6R)	Phase 1	NCT01637532
Siltuximab (CNTO 328)	IL-6R	Phase 2	NCT00841191
Plerixafor	CXCR4	Phase 1	NCT02179970 NCT03277209
PD 0360324+ cyclophosphamide	Macrophage colony stimulating factor (M-CSF)	Phase 2	NCT02948101
Celecoxib + cyclophosphamide	cyclooxygenases (COX-1 and COX-2)	Phase 2	NCT00538031
Ketorolac	COX-1 and COX-2/GTPase inhibition	Phase 0	NCT02470299
Metformin + paclitaxel and carboplatin	Antidiabetic medication/metabolism	Phase 1 Phase 2	NCT02312661 NCT02437812
Metformin	Antidiabetic medication/metabolism	Phase 2	NCT01579812
Metformin+ atorvastatin + doxycycline+ mebendazole	Antidiabetic medication/metabolism (glucose and lipid levels)	Phase 3	NCT02201381
INCAGN01876 + Nivolumab + Ipilimumab	Tumor necrosis factor α (TNFα), Programmed cell death protein 1 (PD-1) and cytotoxic T-lymphocytes’ CTLA-4.	Phase 1 Phase 2	NCT03126110
MK-3475 (pembrolizumab) + Gemcitabine and cisplatin	PD-1	Phase 2	NCT02608684
Oregovomab and Nivolumab	Cancer Antigen 125 (CA-125) and PD-1	Phase 1 Phase 2	NCT03100006
Durvalumab (MEDI4736 + motolimod) + pegylated liposomal doxorubicin	Programmed cell death ligand 1(PD-L1) and Toll like receptor 8 (TLL8)	Phase1 Phase 2	NCT02431559
Autologous Monocytes + Sylatron (PegIFNα)+ Actimmune (IFNγ-1b)	Immunotherapy	Phase 1	NCT02948426
Vigil bi-shRNA furin and GMCSF (FANG) Augmented Autologous Tumor Cell Immunotherapy	TGFβ1 and TGFβ2 (tumor)+ Immune stimulation	Phase 2	NCT02346747
Vigil (Adjuvant FANG)	TGFβ1 and TGFβ2 (tumor) + Immune stimulation	Phase 2	NCT01309230
Atezolizumab and Vigil	PDL1 and TGFβ1 and TGFβ2 (tumor)	Phase 2	NCT03073525
NK immunotherapy	Combination of Cryosurgery and NK Immunotherapy	Phase 2	NCT02849353
Therapeutic autologous Antigen-Specific CD4+ lymphocytes	Immunotherapy	Phase 1	NCT00101257

### Myeloid-derived suppressor cells (MDSCs)

These represent a heterogeneous population of cells of myeloid origin that, in the steady immature state, are present in the bone marrow, but not in secondary lymphoid organs and lack suppressive activity. The phenotype of MDSCs is Lin^-^HLA^−^DR^-^CD33^+^ or CD11b^+^CD14^−^CD33^+^ and have also been identified within a CD15^+^ population in human peripheral blood [121]. When activated by ROS, reactive nitrogen species (RNS), or arginase, arginase they become potent suppressors of various T-cell functions. MDSCs accumulate in lymphoid organs and in tumors in response to growth factors and inflammatory cytokines/chemokines [122] as well as PGE2 enriched in the OvCa TMDC. Obermajer and colleagues [123] showed that PGE-2 attracts MDSC into ascites of OvCa patients by inducing expression of functional CXCR4 in cancer-associated MDSCs, and plays a role in the production of its ligand CXCL12 [123]. These studies provided a rationale for targeting COX-2, and CXCR4 in cancer therapy [123] (Table 1). In tumor tissues, MDSCs can be distinguished from TAMs by their granulocytic morphology, high expression of arginase, inducible nitric oxide synthase (iNOS), and Gr1 (which are not expressed by TAMs) as well as low expression of F4/80 (expressed by TAMs) [121]. In addition to suppressing T-cell functions, high numbers of immunosuppressive MDSCs in the OvCa TME were identified and were shown to promote and maintain the OvCa stem cell pool by stimulating miR-101 expression and targeting co-repressor CtBP2 in OvCa cells [124].

### Dendritic cells (DCs)

Dendritic cells (DCs) are specialized antigen-presenting mononuclear cells, that in their immature state exhibit phagocytic ability, and when become functionally mature, they produce cytokines and exhibit immunostimulatory capacity [125]. DCs are sensitized after exposure to tumor antigen, migrate to regional lymph nodes, and stimulate the proliferation of naive T cells to initiate the immune response [126]. Based on the expression of cluster of differentiation (CD) markers, DCs exhibit antigen cross-presentation via MHC class I or class II molecules to activate CD8^+^ or CD4^+^ T cells [125]. Increased number of tumor-infiltrating DCs in tumor tissue was correlated with favorable prognosis [126]. In addition, substantial numbers of functional plasmacytoid (tolerogenic) dendritic cells (PDCs) were detected in malignant ascites of patients with OvCa and mechanistically, they significantly induced tumor angiogenesis [125]. The ability of DCs to process and present antigens and stimulate anti-tumor immune response promoted the development of clinical trials using DCs vaccines in which autologous DCs pulsed with either tumor cell lysates in combination with bevacizumab and oral metronomic cyclophosphamide for patients with recurrent stage III/IV OvCa in whom anti-tumor responses and clinical benefit for patients were observed [127, 128].

### Lymphocytes

Tumor associated lymphocytes (TILs) comprise T-cells, and regulatory T cells (T-regs) that have left the intravascular compartment and localized in tumor stroma (stromal TILs) or inside the tumor islets (intraepithelial TILs). In particular, intraepithelial TILs play an extensive role in the control of tumor growth in almost all solid tumors including OvCa (summarized in [129]). CD8^+^ or CD4^+^T-lymphocytes can recognize cancer antigens or over-expressed self-antigens and inhibit cancer development [129]. TILs play a key role in tumor immune surveillance through T cell receptor (TCR)-mediated recognition of tumor antigens that have been processed by antigen presenting DCs [130]. Upon recognition of tumor antigens by TCR/MHC engagement, activated CD8^+^ cytotoxic T cells are able to directly kill malignant cells by mechanisms including perforin/granzyme secretion and FasL/Fas binding [131]. Studies indicated that the expression of the death Fas ligand (FasL/CD95L) was exclusively expressed in tumor vasculature and created a barrier that suppressed normal T cell function, allowing tumor cells to grow unrecognized by the immune system. Blocking this selective FasL expression may lead to effective immunotherapy targeting tumor progression [132–134]. Along with CD4^+^ helper T cells, cytotoxic CD8^+^ T-cells can secrete various cytokines/chemokines to direct the activities of other immune cells [135]. Several clinical studies in OvCa, reported a positive correlation between patient survival and the presence of intra-epithelial TILs [135–137]. CD3^+^ TILs were reported in treatment-naïve OvCa specimens, but survival advantage was associated only with intraepithelial but not stromal TILs [135, 138, 139]. Meta-analysis of the majority of reports that investigated the prognostic value of TILs in OvCa [140] using the CD8^+^ marker to specifically evaluate cytotoxic T cells, found that intraepithelial CD8^+^ TILs exhibited a consistent and stronger association with patients’ survival than CD3^+^ TILs [140].

In the inflammatory OvCa TME, TILs’ function is suppressed by regulatory T cells (Tregs), MDSCs, and TAMs, with their secreted plethora of soluble inhibitory factors as IL-6, IL-10, arginase (Arg)1, and TGFβ, various metabolites like adenosine, and depleted tryptophan due to increased indoleamine 2,3-dioxygenase 1 (IDO-1) activity [141, 142]. Suppression of T cell functions occurs through downregulation of MHC molecules and co-stimulatory ligands, with upregulation of inhibitory receptors like programmed cell death protein ligand 1 (PD-L1) on tumor cells and cytotoxic T-lymphocyte antigen-4 (CTLA-4, CD152) [143]. Hamanishi and colleagues [144] reported that high expression of programmed death 1 (PD-1/CD279) on OvCa cells was associated with poorer patients’ survival and with reduced CD8^+^ TILs suggesting that PD-L1 expression promotes an immunosuppressive TME by inhibiting T-cell infiltration [144]. These observations promoted clinical trials targeting PD1 or PDL-1 as well as CTLA-4 in OvCa (Table 1). The efficacy of single or dual blockade of PD-1 and/or CTLA-4 in combination with standard of care therapy was demonstrated in multiple models of OvCa with synergistic effects [143, 145, 146]. However the efficacy in treatment of OvCa patients is still being evaluated in clinical trials.

Regulatory T-cells (Treg) cells. CD4 Tregs are T-cell subpopulation that suppresses the function of activated T-cells. They can be divided into naturally occurring thymus-generated T-regs with a phenotype of CD4^+^CD25^+^FOXP3^+^ and the adaptive Tr1 Treg and Th3 Tregs which have variable CD25 expression. Moreover, patients with OvCa expressed Treg subsets with upregulated cytotoxic T-lymphocyte-associated protein 4 (CTLA-4) and downregulated expression of CD28 [147, 148]. Additionally, *in vitro* induced CD8 Tregs block CD4 T-cells proliferation via TGF-β1 and IFN-γ. Tumors are not only known to increase the number of Tregs in peripheral blood of OvCa patients, but also recruit and stimulate Treg tumor infiltration and localization [147, 148].

Natural killer cells (NK) are lymphocytes of the innate immune system and are defined by the expression of cell adhesion markers CD56 and CD16 and the lack of T-cell receptor CD3 [149]. They target cells with low MHC Class-I expression including tumor cells by using perforins to puncture the membranes of target cells leading to activation of the apoptotic cascade by granzymes. In addition to the aforementioned mechanism, members of the tumor necrosis factor receptor family such as Fas/CD59 also contribute to NK cytotoxicity when activated [150]. In the face of such immune-surveillance, tumor cells have adeptly managed to go undetected via several mechanisms. For instance, studies showed that MUC16, a high molecular weight mucin overexpressed by OvCa has the ability to inhibit NK cell and downregulate CD16. NK cells lose their CD16 by a metalloprotease called ADAM17. Inhibition of this metalloprotease enhances CD16-mediated NK cell killing ability through antibody-mediated cellular cytotoxicity by maintaining the CD16 on the cell surface [151]. This phenomenon is not exclusive to OvCa cells; some leukemia cell lines inhibit NK cells by up-regulating MHC Class-I expression which sends an inhibitory signal to NK cells [150].

Endothelial cells: In the ovarian TME, two factors are critical to modulate the blood vessel structure: cellular permeability and angiogenesis [152]. In normal tissue, the endothelial cells are composed of a single layer of continuous uniform cells with few cytoplasmic projections, while tumor endothelial cells are of abnormal shape and size [152, 153]. OvCa cells secrete a plethora of factors to induce phenotypic changes in the omental microvasculature. Initially identified as vascular permeability factor [154], vascular endothelial growth factor (VEGF) has been long considered as the key regulator of angiogenesis that drives endothelial cell survival, proliferation, and migration while increasing vascular permeability [155]. VEGF is not only produced by cancer cells, but it is also produced by TAMs, CAAs, as well as CAFs [155, 156]. VEGF contributes to the development of peritoneal carcinomatosis associated with malignant ascites formation, the hallmarks of advanced OvCa [156, 157]. Preclinical and clinical studies showed that VEGF levels inversely correlates with disease prognosis and patients’ survival [155–157]. VEGF inhibition has been shown to inhibit tumor growth, invasion, metastasis, and ascites production. These findings promoted the clinical evaluation of agents targeting VEGF/VEGFRs with approval of anti-VEGF humanized antibody (Avastin, bevacizumab) in patients with OvCa [156] as single agents or in combination with standard of care therapy (https://www.cancer.gov/about-cancer/treatment/drugs/ovarian). In addition to VEGF, deregulation of normal endothelium in the peritoneal TME is also induced by proangiogenic factors such as IL-6, IL-8, PDGF, FGF, CCL2, CXCR4, uPA/uPAR, angiopiotein-1, bioactive lipids and neuroendocrine hormones produced by OvCa cells and the other cellular components in the peritoneal TME [120, 156]. This upregulation of the proangiogenic factors and their interconnected signaling pathways not only contributes to increased vascular permeability, tumor growth and angiogenesis, but also contributes to the suboptimal response to VEGF/VEGFR targeting therapy [120, 156]. Therefore, clinical trials targeting these proangiogenic factors, their receptors including receptor tyrosine kinases in OvCa patients, are currently underway (Table 1).

Ascitic fluid. In normal physiologic conditions, the movement of proteins from the intravascular space to the peritoneal fluid is tightly regulated by 5 layer-barrier namely, capillary endothelium, capillary basement membrane, interstitial stroma, mesothelial basement membrane and mesothelial cells of the peritoneal lining [158]. This barrier is maintained by intact tight junctions (at the mesothelial and endothelial interface) and intravascular anionic macromolecules which create a difference in oncotic pressure across the peritoneal membrane [158]. In patients with OvCa, this barrier is breached with increased vascular and mesothelial permeability and transudation of high protein fluid from intravascular compartment to peritoneal cavity. Along with the high protein concentration, increased inflammatory cytokines and chemokines and reduced lymphatic flow also contribute to the buildup of ascitic fluid [6, 25, 156, 158, 159]. In addition, ascites is rich in bioactive lipids as lysophosphatidic acid (LPA), that has been long identified as OvCa promoting factor [160]. LPA is produced by OvCa cells and the cellular components in the peritoneal TME. High levels of LPA in ascitic fluid lead to aberrant receptor signaling and activation of pro-inflammatory and pro-survival pathways as well as transactivation of receptor tyrosine kinases [161–164] that further OvCa progression and are associated with poor prognosis [7, 160, 165]. At the cellular level, ascitic fluid contains floating cancer cells, macrophages and immune cells; all contribute to the malignant aggressive phenotype of OvCa [120, 155–157].

### Treatment

Standard treatment of OvCa is composed of initial surgical management with exploratory laparotomy followed by total hysterectomy, bilateral salpingo-oophrectomy, omentectomy, and retroperitoneal lymphadenectomy depending on the extent and spread of the primary tumor. Initial treatment options are primary surgery followed by chemotherapy and neoadjuvant chemotherapy followed by surgery. Standard chemotherapy involves carboplatin and paclitaxel. Various targeted therapies are being studied in combination with carboplatin/paclitaxel in hopes of improving survival (Table 1). In addition to the recently FDA approved targeted therapies as poly (ADP-ribose) polymerase PARP inhibitors and VEGF inhibitors, other targeted therapies currently in clinical trials include other angiogenesis inhibitors (VEGF/VEGFRs, FGFRs, PDGFRα/β), multi-target receptor tyrosine kinase inhibitors (RTKi), cyclooxygenase-2 (Cox-2) inhibitors, and inhibitors of cytokines and their receptors (IL-6/IL-6R, SDF1/CXCR4, M-CSF, TGFβ1/2/3).

In recent years, immunotherapy for advanced stage OvCa was introduced in clinical trials using immune check points inhibitors targeting programmed cell death protein (PD1) and its ligand (PDL1) as well as cytotoxic T-lymphocyte-associated protein 4 (CTL4) with neutralizing antibodies that restore the functions of cytotoxic T-lymphocytes to recognize and eradicate tumor cells. More recently, personalized therapy with autologous tumor and immune cells reprogrammed *ex-vivo* to stimulate the immune system and overcome immune evasion of OvCa cells are currently in clinical trials. However, emerging data suggests limited survival advantages. In addition, targeting tumor metabolism has recently gained more appreciation as evidenced by clinical trials of metformin in advanced HGSC either alone or in combination of standard of care therapy (Table 1).

Despite the changing nature of chemotherapeutic regimens, OvCa resistance and recurrence still remain a common problem mainly due to suboptimal resection of microscopic and/or lesions that cannot be removed due to their site in the peritoneal cavity, tumor heterogeneity, evolution of chemo-resistant tumor cells and the unique site of OvCa spread in the peritoneal cavity.

## CONCLUSIONS

Advanced epithelial OvCa carries the largest burden of disease mortality among all gynecologic malignancies. Although the majority of patients show substantial initial response to first-line therapy (e.g., surgery and combined platinum plus paclitaxel based therapies), almost 70% of patients experience recurrence of their cancer within 18 months. Our ability to effectively treat recurrent OvCa is the single greatest impediment to improve disease outcome. Current challenges in curing patients with OvCa are: 1) late-stage detection for the majority of OvCa 2) suboptimal debulking surgery mostly due to infiltrative nature of the disease, and 3) recurrence of chemo-resistant cancer. Indeed successful treatment of OvCa can be achieved by improving surgical approaches to precisely excise the tumor with minimal residual disease, and enhancing our understanding of the complex interplay of cancer cells within the unique peritoneal TME. The ultimate goals are: 1) identifying and validating diagnostic biomarkers for early stage disease, 2) identifying and validating prognostic biomarkers in the primary tumor that predict response to therapy and recurrence, and 3) targeting the determinants of cancer cell-TME interactions in neoadjuvant or adjuvant settings.
